# The effect of intrathecal dexmedetomidine on the dose requirement of hyperbaric bupivacaine in spinal anaesthesia for caesarean section: a prospective, double-blinded, randomized study

**DOI:** 10.1186/s12871-018-0528-2

**Published:** 2018-06-23

**Authors:** Feng Xia, Xiangyang Chang, Yinfa Zhang, Lizhong Wang, Fei Xiao

**Affiliations:** Department of Anaesthesia, Jiaxing University Affiliated Women and Children Hospital, Jiaxing, Zhejiang China

**Keywords:** Dose-response, Dexmedetomidine, Intrathecal, Bupivacaine, Caesarean section

## Abstract

**Background:**

Studies have shown that dexmedetomidine (Dex) can prolong the duration of analgesia when added to local anaesthetic as an adjuvant in a central or peripheral nerve block. We hypothesized that intrathecal Dex can reduce the ED95 of spinal hyperbaric bupivacaine. Therefore, we conducted this prospective, double-blinded, randomized study to verify our hypothesis.

**Methods:**

Ninety patients were allocated into the Dexmedetomidine group (received bupivacaine + 5 mcg dexmedetomidine) and the Control group (received bupivacaine + the same volume of saline) using a double-blinded and randomized method. The first patient in each group received 5 mg of IT hyperbaric bupivacaine, and the next dose for the following patient was determined by the probability of successful anaesthesia of the previous neighbouring dose. An improved up-down sequence allocated method combined with probit analysis was used to determine the ED95 of intrathecal hyperbaric bupivacaine for the two groups.

**Results:**

The ED95 and 95% confidence intervals (95% CI) of IT hyperbaric bupivacaine of the Dex group and Control group were 8.4 mg (95% CI, 6.5~ 13.8 mg) and 12.1 mg (95% CI, 8.3~ 312.8 mg), respectively. The duration of sensory block was longer in the Dex group than in the Control group (110.3 ± 35.3 vs 67.5 ± 26.2). The duration of analgesia was also longer in the Dex group than in the Control group (224.9 ± 45.4 vs 155.1 ± 31.6). The consumption of postoperative rescued sufentanil was significantly higher in the Control group than in the Dex group.

**Conclusion:**

Intrathecal 5 mcg dexmedetomidine potentiated hyperbaric bupivacaine antinociception by 31% in spinal anaesthesia for patients undergoing caesarean section.

**Trial registration:**

We registered this study in a Chinese Clinical Trial Registry (ChiCTR) centre on Nov 1st 2016 and received the registration number: ChiCTR-IPR-16009699.

## Background

Spinal anaesthesia is an ideal choice for caesarean section when there are no contraindications to this technique [1]. Hypotension, which is closely related to maternal and neonatal morbidity and mortality, is the most common side-effect of spinal anaesthesia. Studies have proven that lowering the intrathecal local anaesthetic can decrease the incidence of spinal-induced hypotension [2–4]. However, shortcomings that were associated with this technology included a relatively short duration of anaesthesia and analgesia [5]. To overcome this disadvantage, many kinds of adjuvants (fentanyl, sufentanil, epinephrine, etc.) were suggested to help prolong anaesthesia and analgesia [6–9]. However, these adjuvants were associated with undesired side effects [10–14]. Although magnesium sulphate was reported to prolong the duration of spinal analgesia without additional side effects, it failed to reduce the dose requirement of intrathecal bupivacaine in our previous study [15].

Dexmedetomidine (Dex), as an adjuvant, can extend the duration of analgesia of local anaesthetic in spinal, paravertebral nerve and transversus abdominis plane blocks [16–21]. We hypothesized that intrathecal Dex could reduce the value of ED95 (95% effective dose) of spinal hyperbaric bupivacaine. In this study, we aimed to determine the ED95 of intrathecal hyperbaric bupivacaine with or without Dex as an adjuvant in spinal anaesthesia for caesarean section using an improved up-down sequential allocation method.

## Methods

After approval by the Institutional Ethics Committee of Jiaxing University Affiliated Women and Children Hospital and written informed consent from all patients, 90 parturients with the statue of American Society of Anaesthesiologists’ physical class I or II, scheduled for elective caesarean section, were enrolled in this study. Exclusion criteria included the following: gestational age less than 36 weeks, active or early labour, ruptured membranes, placenta previa, patients’ body mass index (BMI) > 35 kg/m^2^, hypertension or pre-eclampsia, diabetes or gestational diabetes, intrauterine growth restriction, history of more than one previous caesarean delivery, and any contraindications to regional anaesthesia, such as local infection or bleeding disorders. We registered this study in a Chinese Clinical Trial Registry (ChiCTR) centre and received the registration number: ChiCTR-IPR-16009699 (URL: http://www.chictr.org.cn/edit.aspx?pid=16461&htm=4).

Ninety healthy parturients were randomly assigned to the Dex group (*n* = 45) and the Control group (*n* = 45), using a random number list generated by computer (Microsoft, Excel). The number list (prepared by F. Xiao, who knew the patient’s group) was stored in a non-transparent envelope before the beginning of this clinical trial.

No participants received premedication. After arriving in the operating theatre, all patients’ peripheric vein was punctured with an 18G puncture needle, and 37 °C *Lactate Ringer’s solution* was injected slowly to keep the vein open before the induction of spinal anaesthesia. Patients’ electrocardiogram (ECG), non-invasive blood pressure (NIBP), heart rate (HR), and oxygen saturation (SpO_2_) were checked and recorded. The average of the first three readings was considered the basal NIBP and HR.

With the parturients in the left lateral position, the combined spinal-epidural anaesthesia (CSEA) was performed using the needle-through-needle technique. After the interspace of L3–4 was estimated, the epidural space was ascertained with the loss-of-resistance-to-air technique (air volume < 2 ml) using an 18-G Tuohy needle. Then, a 27-G spinal needle was passed through the Tuohy needle to reach the subarachnoid space. When the flow of the cerebrospinal fluid (CSF) was observed, the mixed study solution was administered via the spinal needle over 10 s. Before removing the spinal needle, the CSF was withdrawn again to make sure that the drug was injected into the subarachnoid space. If CSF was not withdrawn, the subject was excluded from the study. The anaesthesiologist removed the spinal needle and inserted an epidural catheter into the epidural space for 3–4 cm. No local anaesthetic was given through the epidural catheter at the time. With a 15-degree tilt to the left side, the patients received 500 mL *Lactate Ringer’s solution* as a co-load over 20 min.

The study solution was prepared under sterile conditions in advance by a fixed anaesthesiologist (F. Xiao) who was not involved in assessing the effect of anaesthesia. The CSEA technique was performed by two attending anaesthesiologists (X.Y. Chang and Y.F. Zhang) who were blinded to the patients’ grouping. The study solution for the two groups was 0.75% hyperbaric bupivacaine mixed with 5 mcg of Dex (Dex Group) (Jiangsu Henrui Medical Company, LTD China; Production batch: 16090232) or saline (Control Group) and was diluted to 3 mL with saline. A volume not greater than 1 mL was extracted using an insulin syringe (1 ml).

The dose of spinal bupivacaine for each patient was determined by the modified up-down method [22]. The first parturient in each group received 5 mg hyperbaric bupivacaine. The intrathecal dose of bupivacaine for the following patient was decided by the probability of successful anaesthesia of the previous dose. For example, if the probability of successful anaesthesia of the previous dose of (n) mg exceeded 95%, the next patient in this group would receive (n-1) mg of hyperbaric bupivacaine. Conversely, if the probability of successful anaesthesia was less than 95%, the next patient in this group would receive (n + 1) mg of hyperbaric bupivacaine. If the probability of the dose was 95%, the same dose of intrathecal bupivacaine was applied for the next subject. Successful anaesthesia was regarded as a bilateral T_5_ or higher sensory block level obtained within 10 min after intrathecal injection with no epidural supplement during surgery. Otherwise, the case was regarded as an unsuccessful anaesthesia, and an epidural supplement of 5 mL of 2% lidocaine was given to induce spinal anaesthesia or rescue intraoperative pain, repeated at 5-min intervals if necessary.

The primary outcome of this study was successful anaesthesia or unsuccessful anaesthesia. The secondary outcomes of this study were the characteristics of spinal anaesthesia, analgesic duration of spinal anaesthesia and side-effects.

Consecutive monitoring of NIBP and HR was performed, and the values were recorded at 2-min intervals in the first 10 min after spinal induction and at 5-min intervals thereafter. Hypotension was defined as a systolic arterial pressure below 90 mmHg or a decrease of more than 20% of basal systolic blood pressure. Hypotension was treated with a bolus of 100 μg of intravenous phenylephrine, repeatedly if necessary. Bradycardia, defined as a heart rate less than 55 beats per min, was treated with 0.5 mg of atropine intravenously.

The sensory block level lost to pinprick was checked gently with an 18-G epidural needle along the medioventral line. The period from the intrathecal injection until a T_10_ sensory block level was achieved was regarded as the onset time. The period from the onset time to 2-segment regression of the sensory block was regarded as the duration of the sensory block. The Bromage Score [23] was used to evaluate the motor block level of the lower limbs (0 = can lift leg; 1 = can bend the knees; 2 = can move foot; 3 = cannot move foot). The period from the intrathecal injection to a Bromage Score of 1 was regarded as the onset time of the motor block. The duration from the intrathecal injection to the first time the patient requires postoperative analgesia was regarded as the duration of spinal analgesia. Postoperative pain was managed with a patient-controlled intravenous analgesia (PCIA) pump, which was set with a bolus of 3 μg of sufentanil and with a 10 min interval of locking time. The sensory and motor block was checked at 1-min intervals during the first 10 min after the intrathecal injection, at 5-min intervals during the surgery, and at 30-min intervals in the obstetric ward before full recovery. After the surgery, patients were required to fill out the satisfaction questionnaire (1 = satisfied; 2 = moderate; 3 = poor).

Side effects such as hypotension, bradycardia, nausea and vomiting, shivering, pruritus, and severe sedation were recorded and studied. Complications of spinal anaesthesia such as post-dural headache (PDPH) and any symptoms and signs of neurological deficits were also recorded and studied. Sedation was ranked as none = awake and alert, mild = awake but drowsy, moderate = asleep but arousable, and severe = not arousable. The pH value of the umbilical arterial blood that was drawn immediately after infant delivery was assessed as the outcome of the infant. After a month of surgery, all patients received a telephone follow-up that was mainly about neurological deficits.

### Statistical analysis

According to previous study conducted by M. Tanaka et al. [24], a sample size of 45 patients for each group was determined, after testing a variety of scenarios, each with a thousand simulations of both the responses and the corresponding doses selected by the up-down method described in the methods section in this study and beginning with various starting doses. According to our preliminary experiment, to detect a difference of 3 mg in the dose requirement of intrathecal bupivacaine (ED95) with an α error of 0.05 and test power of 90%, at least 39 patients for each group were needed.

Demographic data, duration of surgery, onset time to T_10_, onset time to the motor block, onset time to the highest block level and duration of the sensory block and analgesia were described as the mean ± SD and tested with Student’s *t* test. The highest sensory block level was presented as a median (range) and tested with Mann-Whitney U test. The degree of the patient satisfaction and side effects were described as a number (rate) and were analysed with Chi-square test. Kaplan-Meier survival analysis was used to analyse the duration of spinal analgesia. GraphPad Prism 5 was used to perform the statistical analysis. A *P* value less than 0.05 was regarded as a significant difference (two-sided).

## Results

**Fig. 1:**
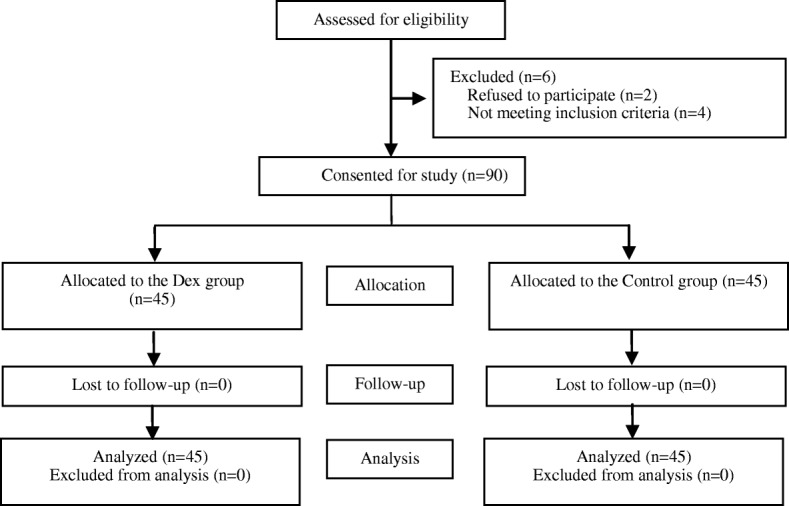
Consort

The CONSORT diagram is shown in Fig. 1. This clinical trial was initiated on November 10, 2016, and was finished on May 1, 2017. During this period, 96 parturients were involved and assessed for suitability for this clinical trial. Finally, 90 parturients were enrolled and allocated to the two groups. None of the 90 parturients was lost in the final analysis.

There was no significant difference in demographic data, obstetric data or duration of surgery between the two groups (Table 1).

**Table 1:** Patient demographic, obstetric and surgical data

	Dex group (*n* = 45)	Control group (*n* = 45)	*P-*value^a^
Age (y)	26 ± 3	25 ± 4	0.43
Height (cm)	164 ± 3	163 ± 3	0.41
Weight (kg)	73 ± 4	72 ± 3	0.84
Gestational age (week)	39 ± 1	39 ± 1	0.60
Duration of surgery (min)	44 ± 7	46 ± 8	0.42

Data are presented as the mean ± SD

^a^Student *t* test

**Fig. 2:**
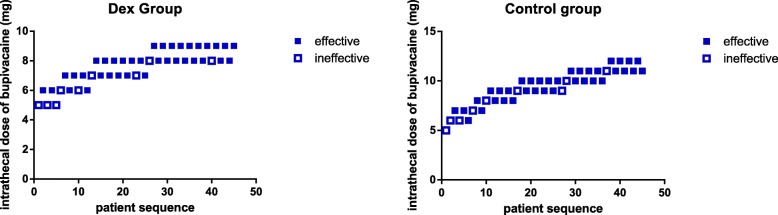
Individual response and dose of intrathecal hyperbaric bupivacaine

**Fig. 3:**
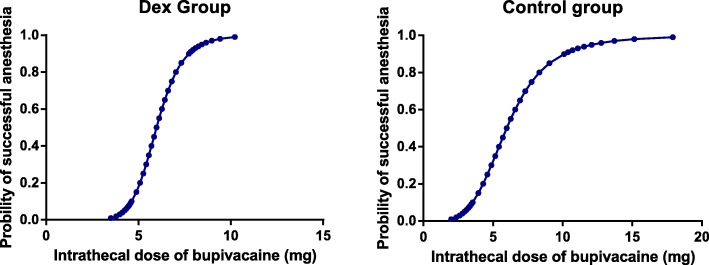
Dose-response curve of intrathecal hyperbaric bupivacaine in the two groups

The ED95 and 95% confidence intervals (CIs) of intrathecal hyperbaric bupivacaine in the Dex group and Control group were 8.4 mg (95% CI, 6.5~ 13.8 mg) and 12.1 mg (95% CI, 8.3~ 312.8 mg), respectively. The ED95 of intrathecal hyperbaric bupivacaine was lower in the Dex group than in the Control group. Intrathecal 5 μg Dex can decrease the ED95 of hyperbaric bupivacaine by 31%. The subsequent response of each dose of intrathecal hyperbaric bupivacaine (effective or ineffective) is shown in Fig. 2. The dose-response curves of IT hyperbaric bupivacaine in the two groups are shown in Fig. 3.

**Fig. 4:**
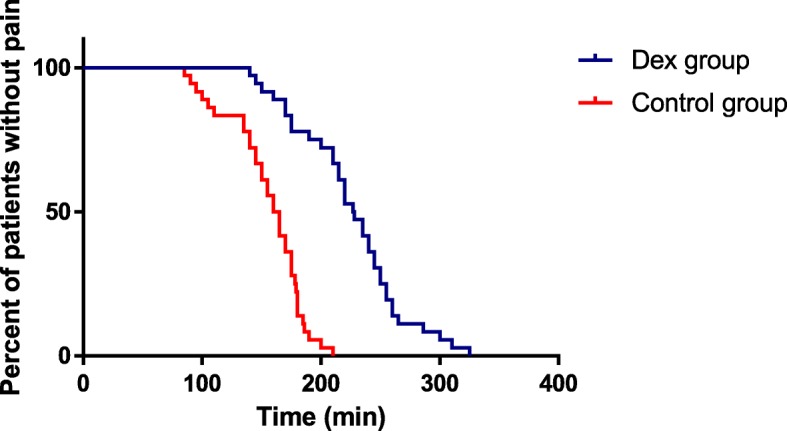
Duration of spinal anaesthesia

Characteristics and efficacy of spinal anesthesia in patients with “effective anesthesia” are presented in Table 2. The onset time to T10 was similar in the two groups (3.8 ± 1.1 vs. 3.6 ± 1.3 min, *P*>0.05). The onset time to motor block was also similar in the two groups (3.8 ± 2.1 vs. 3.4 ± 1.9, *P*>0.05). There was no significant difference in the highest block level between the two groups [(T5 (T3-T6) vs. T5 (T3-T6), *P*>0.05], and the time to the highest block level was also similar (13.7 ± 4.8 vs. 11.7 ± 4.0, *P*>0.05). There was a significant difference in the duration of the sensory block between the Dex group and the Control group (110.3 ± 35.3 vs. 67.5 ± 31.2, *P*<0.05). The duration until patients required their first postoperative analgesic was longer in the Dex group than in the Control group (224.9 ± 45.4 vs. 155.1 ± 31.6, *P*<0.001) (Fig. 4). The total requirement of postoperative rescued sufentanil during the first 24 h in the Dex group was less than in the Control group (48.6 ± 9.8 vs. 64.8 ± 11.8, *P*<0.05). There was no significant difference in the patient satisfaction of analgesia between the two groups (*P*>0.05).

**Table 2:** Characteristics of spinal anaesthesia in patients with effective anaesthesia

	Dex group (*n* = 36)	Control group (*n* = 36)	*P-*value
Sensory block (to pinprick)			
Highest level of block	T5 (T3-T6)	T5 (T3-T6)	>0.05^a^
Onset time to T_10_ (min)	3.8 ± 1.1	3.6 ± 1.3	>0.05^b^
Time to highest level	13.7 ± 4.8	11.7 ± 4.0	>0.05^b^
Duration (min)	110.3 ± 35.3	67.5 ± 31.2	<0.05^b^
Motor block			
Onset time (min)	3.8 ± 2.1	3.4 ± 1.9	>0.05^b^
Duration of analgesia (min)	224.9 ± 45.4	155.1 ± 31.6	<0.001^b^
Consumption of sufentanil (μg)	48.6 ± 9.8	64.8 ± 11.8	< 0.05^b^
Patient satisfaction			
Excellent [number (%)]	22 (61)	19 (53)	>0.05 ^c^
Good [number (%)]	14 (39)	17 (47)	>0.05 ^c^

Data are presented as the mean ± SD or number (%)

^a^Mann-Whitney,^b^Student *t* test, ^c^Chi-square test

There was no significant difference in the incidence of side effects and neonatal umbilical arterial pH, which is shown in Table 3. There were no reports from patients about neurological deficits during the first month after surgery in the two groups.

**Table 3:** Side effects of anaesthesia, neonatal Apgar score and umbilical arterial pH

	Dex group (*n* = 45)	Control group (*n* = 45)	*P* –Value
Hypotension	8 (18)	15 (33)	0.09^b^
Nausea and vomiting	8 (18)	7 (16)	0.78^b^
Shivering	7 (16)	9 (20)	0.58^b^
Pruritus	0	0	1^b^
PDPH	1 (2)	0	0.32^b^
Severe sedation	0	0	1^b^
Respiratory depression	0	0	1^b^
Umbilical artery pH	7.28 ± 0.04	7.27 ± 0.05	0.26^a^

Data are presented as a number (%) or mean ± SD

^a^Student’s *t* test, ^b^Chi-square test

## Discussion

In the current study, we chose 5 mcg Dex as an IT adjuvant of bupivacaine based on several previous studies in which authors reported that IT 5 mcg Dex can extend the duration of spinal analgesia without any additional side effects [18, 20, 24]. Considering that a caesarean section is a swift procedure, and according to our previous study, [15] we defined effective anaesthesia as a bilateral T_5_ or above sensory block level achieved within 10 min of IT drug administration with no additional epidural anaesthetic required for intraoperative pain.

We found that the ED95 of bupivacaine was 8.4 mg (95% CI, 6.5~ 13.8 mg) in the Dex group and 12.1 mg (95% CI, 8.3~ 312.8 mg) in the Control group. IT 5 mcg Dex can decrease the ED95 of IT hyperbaric bupivacaine by 31% in parturients undergoing caesarean section. To the best of our knowledge, this is the first publication in which IT 5 mcg Dex was found to decrease the ED95 of IT hyperbaric bupivacaine. We also demonstrated that the duration of sensory block and analgesia were prolonged and the consumption of rescued sufentanil was reduced by IT 5 mcg Dex. This was similar to several previous studies. *Samantaray* et al. [20] reported that, with the addition of IT 5 mcg Dex, the time to the first rescued analgesic request was prolonged by nearly 120 min, and the analgesic requirement was reduced in 24 h compared to adding saline or midazolam. In a dose-response trial, the authors found a dose-related extension of analgesia with the addition of Dex. [25] *Qi* et al. [26] also demonstrated that Dex was similar to morphine, prolonged analgesic duration and reduced the incidence of side-effects.

Dexmedetomidine, a highly selective, alpha-2-adrenergic receptor (α2-AR) agonist, has been popularly used by anaesthetists in various anaesthetic techniques due to its haemodynamic-stabilizing properties and sedative, analgesic, and sympatholytic effects [27, 28]. There were three possible mechanisms to explain the enhanced anaesthetic efficiency and prolonged duration of postoperative analgesia in this study. First, some researchers believe that Dex, via the action of α2-AR, induces vasoconstriction, which might contribute to prolonging the period of analgesia. [29, 30] Eledjam et al. [31] added clonidine and epinephrine to local anaesthetics and demonstrated that clonidine plays a role through α2-AR agonists rather than through vasoconstriction. Similar to clonidine, Dex may work via α2-AR agonists. Later, *Yoshitomi* et al. [32] suggested that Dex may enhance local anaesthetic action by the action of α2-AR in a pig study. It might be expected that Dex potentiates the spinal block via a synergistic interaction between α2-AR antagonists and sodium channels, resulting in a reduction in the dose of the local anaesthetics required for achieving effective spinal anaesthesia for certain surgical procedures.

There were several studies concerning the safety of Dex as an adjuvant in spinal anaesthesia. A preclinical study demonstrated that adding Dex to ropivacaine extends the duration of the sensory blockade but showed no neurotoxicity, even at a high-dose of 20 μg/kg of Dex administered with ropivacaine in sciatic nerve blocks in rats [33]. In another animal study, the author found that IT Dex can produce antinociception without any histopathological signs of injury in the spinal cord in rats [34]. Moreover, *Goyagi* et al. [35] reported that continuous infusion of intravenous Dex can improve neurological and histological outcomes 48 h after transient spinal ischaemia in rats. In our clinical practice, no reports suggested any neurological deficit associated with intrathecal Dex [18, 20, 25]. No abnormal symptoms or signs in the nervous system were found, which suggest that Dex is a safe intrathecal adjuvant. Side effects between the two groups were similar in this study. However, the incidence of hypotension in the Dex group was slightly lower than in the Control group, which suggested that lowering an intrathecal local anaesthetic can decrease the incidence of spinal-induced hypotension [2–4].

Limitations existed in the current study. First, we only observed one dose of IT Dex. Further studies should focus on whether a further increase in the dose of IT Dex can decrease the ED95 of spinal bupivacaine and subsequently decrease the incidence of hypotension. Second, we did not observe the duration the motor block. However, the primary purpose of this study was to determine the ED95 of IT bupivacaine. Third, the IT application of Dex was off-label use. Further studies using large, multicentre populations are needed to determine the safety of IT Dex.

## Conclusions

Intrathecal 5 mcg dexmedetomidine potentiated hyperbaric bupivacaine antinociception by 31% in spinal anaesthesia for patients undergoing caesarean section and prolonged the spinal analgesia duration without additional side effects.

## Abbreviations

BMI: body mass index
CSEA: combined spinal-epidural anaesthesia
CSF: cerebrospinal fluid
Dex: dexmedetomidine
ECG: electrocardiogram
ED95: 95% effective dose
HR: heart rate
IT: intrathecal
NIBP: non-invasive blood pressure
PCIA: patient-controlled intravenous analgesia
PDPH: post-dural puncture headache
SpO_2_: saturation
